# Stereotactic body radiation therapy combined with cadonilimab and lenvatinib in the treatment of hepatocellular carcinoma with Vp3 or Vp4 portal vein tumor thrombus: a prospective, multicenter, single-arm, phase II clinical trial

**DOI:** 10.3389/fimmu.2025.1687344

**Published:** 2025-11-10

**Authors:** Ning Mo, Kai Hu, Zhiming Zeng, Lihua Yang, Yeying Fang, Jie Zeng, Ye Li, Zhendong Yang, Jing Tang, Tingting Zhang, Fuchao Ma, Cuizhen Liu, Haiping Zheng, Jinfeng Qiu, Yanfeng Jiang, Yufeng Lv, Li Liang, Xiaofang Huang, Xiwen Liao, Yan Wang, Xiuyuan Lu, Lijun Ning, Shixue Lao Guo, Jie Ma, Rensheng Wang

**Affiliations:** 1 Department of Oncology, The First Affiliated Hospital of Guangxi Medical University, Nanning, Guangxi, China; 2 Department of Radiotherapy, The First Affiliated Hospital of Guangxi Medical University, Nanning, Guangxi, China; 3 Department of Oncology, Foresea Life Insurance Guangxi Hospital, Nanning, Guangxi, China; 4 Department of Oncology, The Second Affiliated Hospital of Guangxi Medical University, Nanning, Guangxi, China; 5 Department of Radiology, The First Affiliated Hospital of Guangxi Medical University, Nanning, Guangxi, China; 6 Department of Hepatobiliary Surgery, The First Affiliated Hospital of Guangxi Medical University, Nanning, Guangxi, China; 7 Medical Center, Akeso Biopharma, Inc, Zhongshan, China; 8 Guangxi Medical University, Nanning, Guangxi, China

**Keywords:** hepatocellular carcinoma, portal vein tumor thrombus, cadonilimab, lenvatinib, stereotactic body radiation therapy

## Abstract

**Background:**

Hepatocellular carcinoma (HCC) patients with portal vein tumor thrombus (PVTT) exhibit a dismal prognosis, occurring in 44%–62.2% of cases at initial diagnosis. The optimal treatment for this population remains undefined.

**Methods:**

In this prospective, multicenter, single-arm phase II trial across three Chinese centers, eligible HCC patients with Vp3/4 PVTT received combined stereotactic body radiotherapy (SBRT), cadonilimab, and lenvatinib. Primary endpoint was objective response rate (ORR) in primary liver lesions (RECIST v1.1/mRECIST); secondary endpoints included ORR in PVTT, progression-free survival (PFS), overall survival (OS), duration of response (DOR), and safety.

**Results:**

Of 24 enrolled patients, 21 were evaluable for efficacy. ORR for primary lesions was 38.1% (RECIST v1.1) and 47.6% (mRECIST), with a disease control rate (DCR) of 100% by both criteria. For PVTT, ORR and DCR were 76.2% and 100%, respectively. At a median follow-up of 19.7 months, median PFS was 6.8 months (95% CI: 4.6–12.9), DOR was 10.4 months (95% CI: 2.9–NE), and OS was 13.4 months (95% CI: 6.8–NE). Early biomarker declines (≥75% AFP reduction or ≥50% PIVKA-II decline at 6 weeks) correlated with superior outcomes: AFP reduction predicted longer PFS (HR=0.22, p=0.006) and OS (HR=0.25, p=0.024); PIVKA-II reduction similarly predicted PFS (HR=0.28, p=0.007) and OS (HR=0.18, p=0.002). Common treatment-related adverse events (TRAEs) included hypertension (50%), thrombocytopenia (42%), and fatigue (38%).

**Conclusions:**

The combination of SBRT, cadonilimab, and lenvatinib showed promising efficacy and manageable toxicity in HCC patients with Vp3/4 PVTT. Early AFP or PIVKA-II response at 6 weeks may serve as a prognostic biomarker.

**Clinical Trial Registration:**

ClinicalTrials.gov, identifier NCT06040177.

## Introduction

According to 2022 Global Cancer Statistics, hepatocellular carcinoma (HCC) ranks fifth in incidence and third in mortality among all malignancies worldwide (1). Moreover, on the basis of 2022 data from the National Cancer Center of China, in China HCC ranks fourth in incidence and second in mortality among all cancers (2). The absence of early-stage symptoms often leads to delayed diagnosis, with 44–62.2% of HCC patients initially presenting with portal vein tumor thrombus (PVTT) (3). This vascular complication, particularly when involving the right or left portal vein (Rt/Lt PV), main portal vein (MPV), or superior mesenteric vein (SMV) - classified as Vp3 or Vp4 PVTT - is associated with an exceptionally poor prognosis. Patients managed with best supportive care alone demonstrate a median overall survival (OS) of merely 2.2 months (4). Current clinical guidelines from the Barcelona Clinic Liver Cancer (BCLC) staging system recommend systemic therapy, including sorafenib (5) and lenvatinib (6), as the standard approach for such cases. The success of drugs in phase III clinical trials, including IMBrave150 (7), ORIENT-32 (8), and CARES-310 (9), underscores the critical role of targeted and immune combination therapies in the first-line treatment of advanced HCC. A subgroup analysis of the IMbrave 150 study indicated that patients with PVTT benefitted more from atezolizumab plus bevacizumab than from sorafenib alone. Notably, in 73 patients with Vp4 PVTT the combination regimen achieved only a 23% ORR, a median progression-free survival (PFS) of 5.4 months and a median OS of 7.6 months (7). A subgroup analysis of the phase III randomized controlled trial LEAP 002 (10) revealed that patients with macrovascular invasion/extrahepatic metastasis, hepatitis B virus (HBV)-related HCC, and alpha-fetoprotein (AFP) levels >400 ng/mL benefited more from pembrolizumab combined with lenvatinib than from lenvatinib alone. Building on recent phase III evidence from the HIMALAYA (11) and CheckMate 9DW trials (12), the FDA has approved two combination immunotherapies for first-line treatment of unresectable HCC: tremelimumab plus durvalumab and nivolumab plus ipilimumab. These approvals demonstrate that dual immune checkpoint inhibition targeting CTLA-4 and PD-1/PD-L1 pathways shows clinically significant efficacy in advanced HCC. However, the nivolumab-ipilimumab regimen demonstrated an increased toxicity profile, with grade 3/4 treatment-related adverse events (TRAEs) occurring in 41% of patients and serious adverse events (AEs) in 28%, representing a significantly higher burden compared to PD-1 inhibitor monotherapy (13).

Cadonilimab is a novel symmetric tetravalent PD-1/CTLA-4 bispecific antibody with a crystallizable fragment (Fc)-null design with transbinding and enhanced target binding avidity. With no binding to Fc receptors, cadonilimab shows minimal antibody-dependent cellular cytotoxicity, antibody-dependent cellular phagocytosis, and interleukin-6 (IL-6)/IL-8 release, all of which may increase its safety in the treatment of cancer. A multicentre, open-label, phase 1b/2 trial revealed that cadonilimab exhibited encouraging tumour response rate, along with a manageable safety profile in advanced HCC (14). Preliminary results from a single-arm, open-label, multicenter phase IB/II clinical trial evaluating cadonilimab combined with lenvatinib as a first-line treatment for advanced HCC have shown promising antitumor effects and a favorable safety profile (15). Despite these advancements, therapeutic outcomes for HCC patients with Vp3/4 PVTT remain suboptimal due to the relatively low proportion of PVTT patients included in these trials, ranging from approximately 15% to 43% (7–10). There is no consensus on the optimal management strategy for HCC with PVTT between Western practices and those in the Asia–Pacific region, where more aggressive locoregional treatments are recommended for certain patients (16, 17).

Accumulating clinical evidence supports the prognostic benefit of combined systemic and locoregional therapy for HCC patients presenting with PVTT (18, 19). Among locoregional therapies, stereotactic body radiotherapy (SBRT) has shown great promise in the local control of PVTT (20). Advances in technology have enabled the delivery of high doses of radiation to the targeted tumor area in fewer fractions via SBRT while minimizing damage to surrounding healthy tissues (21, 22). A systematic review and meta-analysis of 37 studies involving 2,513 patients with HCC and PVTT demonstrated that SBRT was associated with significantly higher response rates and a lower incidence of grade ≥3 complications than three-dimensional conformal radiation therapy (3DCRT) and selective internal radiation therapy (SIRT) (23). Although SBRT can achieve high rates of local tumor control ((18, 24)), many patients experience out-of-field progression, highlighting the need for concurrent systemic disease control (24). The integration of targeted agents and immunotherapy with radiotherapy has been shown to improve outcomes in patients with unresectable hepatocellular carcinoma (25). However, most of these studies were retrospective and no studies have specifically assessed the efficacy of combining SBRT with cadonilimab and lenvatinib. This study aimed to investigate the efficacy and safety of this triple therapy as a first-line treatment for patients with HCC and Vp3/4 PVTT.

## Materials and methods

### Diagnosis and patient selection

Patients with HCC and Vp3/4 PVTT who received cadonilimab and lenvatinib in combination with SBRT from March to December 2023 at the First Affiliated Hospital of Guangxi Medical University, the Second Affiliated Hospital of Guangxi Medical University, and the Foresea Life Insurance Guangxi Hospital were prospectively studied. The diagnosis of HCC was based on biopsy or clinical criteria outlined in the Guidelines for the Diagnosis and Treatment of Primary Liver Cancer (2022 Edition)[27], and PVTT was diagnosed according to the EASL Guidelines and the Chinese Expert Consensus on Multidisciplinary Diagnosis and Treatment of PVTT (26, 27). PVTT staging was performed using Cheng’s classification (28) and the portal vein invasion (Vp) classification (29). After a multidisciplinary consultation, patients deemed suitable for treatment with cadonilimab and lenvatinib in combination with SBRT were fully informed about the potential efficacy of the combined treatment, possible TRAEs, and associated costs.

The key inclusion criteria for patients were as follows: 1. HCC patients with PVTT. PVTT was confirmed using typical radiological patterns on dynamic contrast-enhanced CT or MRI; 2. Aged between 18 and 70 years; 3. Unresectable HCC was assessed by experienced liver surgeons; 4. An Eastern Cooperative Oncology Group (ECOG) performance status score ranging from 0–2. 5. Good liver function was classified as Child–Pugh class A or B (Child–Pugh score of 7); 6. At least one measurable lesion on CT or MRI, as defined by RECIST version 1.1, that had not been previously treated with locoregional therapies; 7. Patients with HBV infection must receive antiviral treatment according to guideline standards. The key exclusion criteria included the following: 1. Contraindications to cadonilimab, lenvatinib, or SBRT; 2. Presence of central nervous system or leptomeningeal metastasis; 3. Risk of gastrointestinal bleeding; 4. Prior systemic therapy for HCC; 5. Histories of other cancers; 6. Pregnancy. This study was conducted in compliance with the ethical standards of the Declaration of Helsinki and was approved by the Ethics Committee of the First Affiliated Hospital of Guangxi Medical University. Informed consent was obtained from all patients prior to treatment and for their data to be used in clinical research. The study was registered with the Clinical Research Registry managed by the National Institutes of Health (NCT06040177).

### Study design

**Figure 1:**
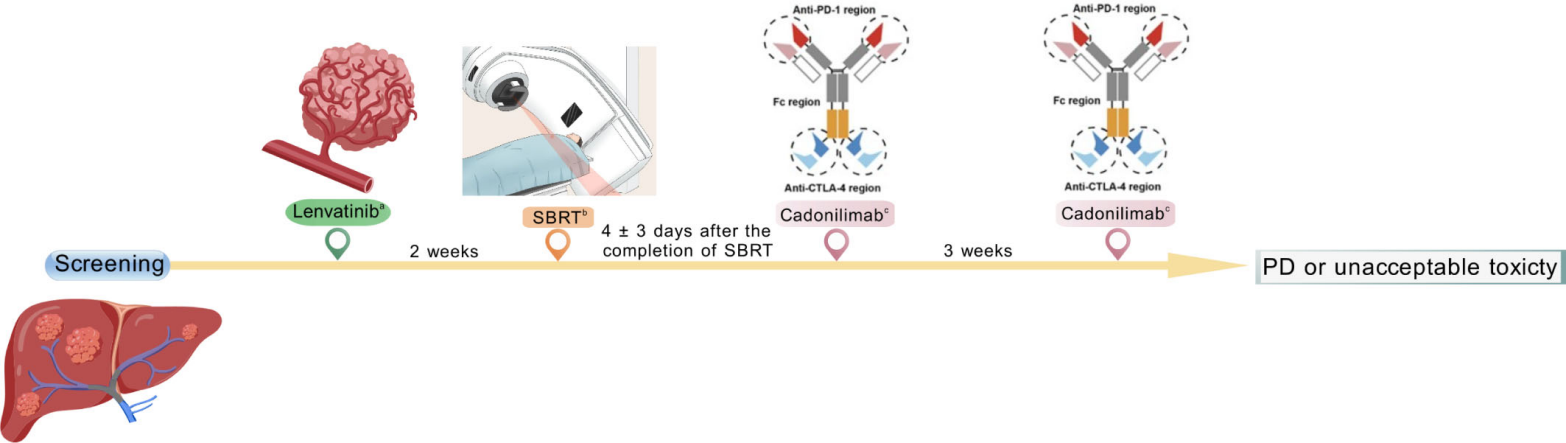
Study design. **(a)** lenvatinib 8 mg (if body weight<60 kg) or 12 mg (if body weight ≥ 60 kg) orally once daily. **(b)** SBRT 30–40 Gy delivered in five fractions. **(c)** Cadonilimab 10mg/kg intravenously Q3W.

The treatment protocol implementation is illustrated in **Figure 1**. After the informed consent form was signed, the patients were administered lenvatinib at a dose of 8 mg daily if their body weight was less than 60 kg or 12 mg daily if it was 60 kg or more. SBRT was planned 14 days after oral lenvatinib administration. Four-dimensional computed tomography (4D-CT) was used in treatment planning to account for respiratory motion. The treatment volume incorporated the PVTT and immediately adjacent tumor tissue (1-cm margin), as identified by dynamic contrast-enhanced cross-sectional imaging, with careful exclusion of synchronous intrahepatic primary lesions from the radiotherapy protocol. The planning target volume (PTV) had a uniform GTV expansion of 5 mm (20). Under the premise of not exceeding the tolerance dose of normal liver tissue, the initial GTV prescription dose was 30–40 Gy delivered in five fractions using 6 MV X-rays with a linear accelerator (23, 30). The cone beam computed tomography (CBCT) data were matched before daily radiotherapy.

Cadonilimab was initiated 4 ± 3 days after the completion of radiotherapy. A fixed dose of 10 mg/kg cadonilimab was administered intravenously on day 1, every 3 weeks (Q3W). Treatment continued until unacceptable toxicity or loss of clinical benefit was achieved, as assessed by investigators on the basis of imaging findings, biochemical parameters, and the patient’s clinical status.

### Outcomes and assessment

Patients were followed up in the outpatient or inpatient department once every 6 weeks (± 7 days) until week 48 and once every 12 weeks (± 7 days) thereafter until death, loss to follow-up, or study completion. Each visit included routine assessments such as medical history review, physical examination, laboratory blood tests (including AFP and protein induced by vitamin K absence or antagonist-II, [PIVKA-II]) and abdominal contrast-enhanced CT/MRI scans. Tumor response was evaluated using RECIST version 1.1 and modified RECIST (mRECIST). For PVTT, partial response (PR) was defined as any downstaging in the PVTT classification or significant restoration of blood flow in the portal vein; progressive disease (PD) was defined as any upstaging in the PVTT classification; and stable disease (SD) was defined as conditions that did not meet the criteria for PR or PD (31).

The primary endpoint was the ORR of measurable target lesions in primary liver tumors, which was defined as the proportion of patients who achieved a confirmed complete response (CR) or PR. The secondary endpoints included ORR of PVTT, PFS, OS, duration of response (DOR), disease control rate (DCR), and safety profile. PFS was measured from the start of treatment to tumor progression, death from any cause, or the last follow-up. OS was calculated from the start of treatment to the date of death or the last follow-up. DOR was defined as the interval from the initial confirmation of a CR or PR to the time of PD or death from any cause. DCR was defined as the percentage of patients who achieved CR, PR, or SD. TRAEs were assessed according to the Common Terminology Criteria for Adverse Events (CTCAE) version 5.0. All patients who received at least one dose of cadonilimab were included in the adverse reaction analysis. For multiple occurrences of the same AE in a patient, the highest grade within that category was recorded. After disease progression, patients receive optimal supportive care on the basis of their general condition, liver function, and extent of HCC. Tumor progression was evaluated using RECIST version 1.1.

### Statistical analysis

Descriptive statistics were used to summarize the baseline patient characteristics. Continuous variables with a nonnormal distribution were compared using the Mann-Whitney U test, whereas categorical variables were analyzed with either the chi–square test or Fisher’s exact test, as appropriate. The tumor response rates are expressed as proportions. Time-to-event endpoints, including PFS, OS, DOR, and were evaluated via Kaplan-Meier methodology with log-rank tests for group comparisons. All the statistical analyses were performed using R version 4.4.0 (2024-04-24) with the Zstats package (version 1.0; www.zstats.net).

## Results

### Patients

**Figure 2:**
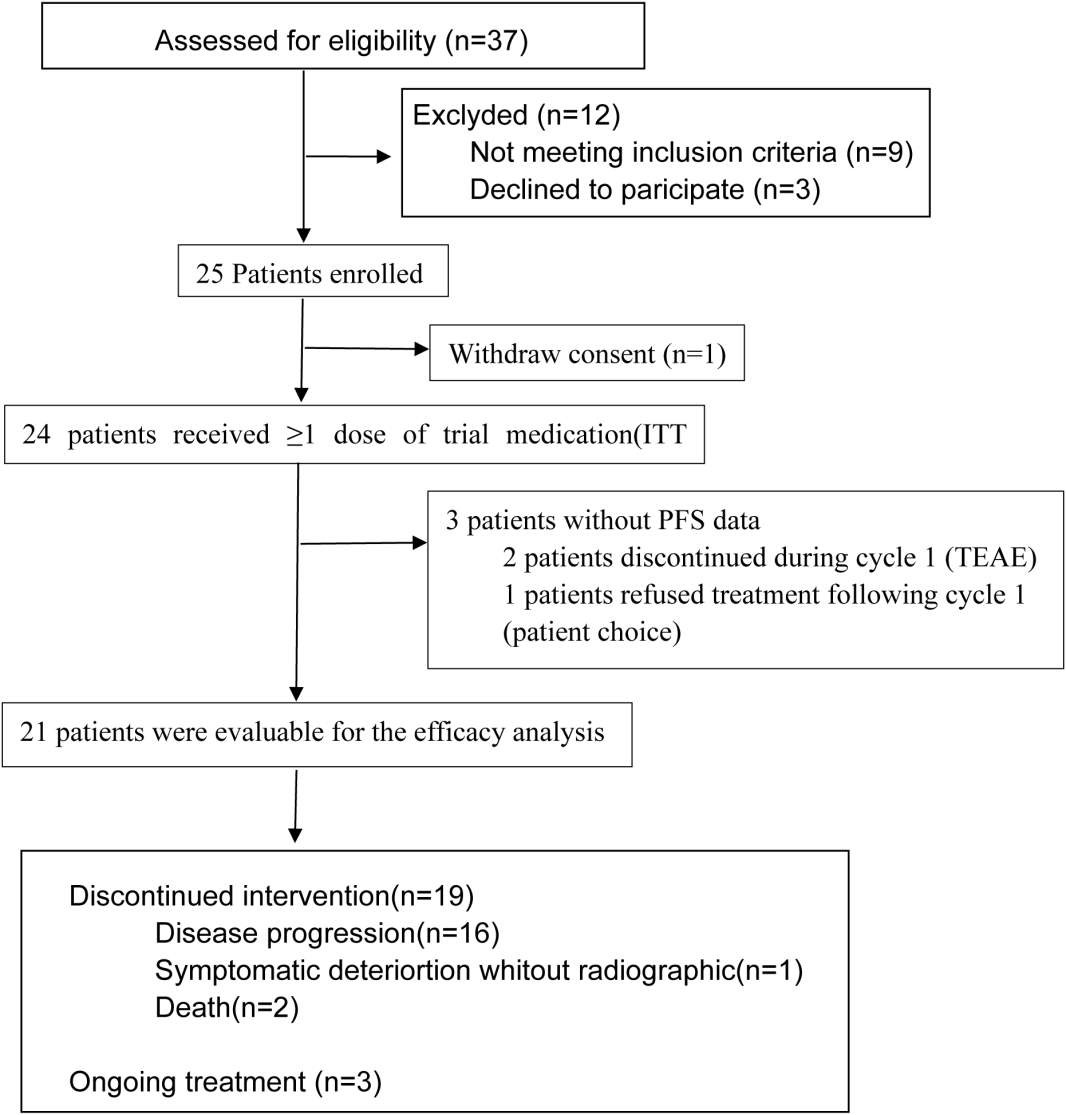
Patient flow diagram.

From March 2023 to December 2023, 37 patients were screened for eligibility; twenty-five patients were enrolled, and the safety population included 24 patients (**Figure 2**). Since patients with HCC and Vp1/Vp2 PVTT typically undergo interventional therapy-based combination treatments, our study ultimately enrolled only patients with Vp3/Vp4 PVTT. A total of 21 patients were included in the final efficacy analysis (**Figure 2**). The baseline clinicopathological characteristics of these patients are summarized in **Table 1**. The median age was 53 years (IQR, 45–58 years), all patients (21, 100%) were coinfected with HBV, and one patient was coinfected with HBV and hepatitis C virus (HCV). Seventeen patients (81%) had cirrhosis, five patients (21%) had an ECOG performance status of 1, and five patients (21%) had Child–Pugh class B (7) liver function. Eleven (53%) HCC patients had Vp3, and ten (47%) had Vp4. Most patients had a maximum tumor size ≥10 cm [13 (62%)] or multiple tumor lesions [17 (81.0%)]. Ten patients (48%) had extrahepatic metastasis, and six patients (29%) had tumors in ≥40% of the liver. During the study period, three of the twenty-four enrolled patients discontinued treatment following cycle 1 (**Figure 2**). Discontinuations were attributed to grade ≥3 TRAEs in two cases (cerebral infarction and upper gastrointestinal hemorrhage, respectively), while the third withdrawal was patient-initiated. On March 28, 2025, when this study was censored, the median follow-up for the entire cohort was 19.7 months, and the median number of cycles of cadonilimab was eight.

**Table 1:** Baseline characteristics of study patients.

Characteristics	All (n=21)	Vp3 (n=11)	Vp4^*^ (n=10)	P
		Cheng’s classification: PVTT type II (n=11)	Cheng’s classification: PVTT type III (n=7) PVTT type IV (n=3)	
Age, years, median (IQR)	53 [45, 58]	49 [45, 53]	57 [47, 66]	0.077
Sex, n (%)				
Male	20 (95)	11 (52)	9 (43)	0.476
Female	1 (5)	0	1 (5)	0.476
ECOG PS, n (%)				
0	7 (33)	4 (19)	3 (14)	0.999
1	13 (62)	6 (29)	7 (33)	0.659
2	1 (5)	1 (5)		0.999
Child-Pugh grade, n (%)				
A	19 (90)	10 (48)	9 (43)	0.999
B (7)	2 (10)	1 (5)	1 (5)	0.999
Cause of diseasea, n (%)				
HBV infection	20 (95)	11 (52)	9 (43)	0.476
HBV and HCV infection	1 (5)		1 (5)	0.476
Liver cirrhosis				
yes	17 (81)	10 (48)	7 (33)	0.311
no	4 (19)	1 (5)	3 (14)	0.311
PVTT classification, n (%)				
Tumor size, maximum, n (%)				
<10 cm	8 (38)	3 (14)	5 (24)	0.387
≥10 cm	13 (62)	8 (38)	5 (24)	0.387
Tumor number, n (%)				
1	4 (19)	3 (14)	2 (10)	0.999
2-5	12 (57)	7 (33)	6 (28)	0.395
>5	5 (24)	1 (5)	4 (9)	0.149
Extrahepatic involvement n (%)				
Yes	10 (48)	3 (14)	7 (33)	0.086
No	11 (52)	8 (38)	3 (14)	0.086
AFP, n (%)				
<400 mg/L	7 (33)	3 (14)	4 (19)	0.635
≥400 mg/L	14 (67)	8 (38)	6 (29)	0.635
PIVKA-II				
< 2050 mAU/mL	2 (10)	1 (5)	1 (5)	0.999
≥2050 mAU/mL	19 (90)	10 (48)	9 (43)	0.999
Tumor of the liver n (%)				
<40%	15 (71)	7 (33)	8 (38)	0.635
≥40%	6 (29)	4 (19)	2 (10)	0.635
Previous locoregional therapy n (%)				
No	21 (100)	11 (52)	10 (48)	0.999

AFP, α-fetoprotein; PIVKA-II, protein induced by vitamin K absence or antagonist-II; BCLC, Barcelona Clinic Liver Cancer; ECOG PS, Eastern Cooperative Oncology Group performance status; HBV, hepatitis B virus; HCV, hepatitis C virus; IQR, interquartile range; PVTT, portal vein tumor thrombus. ^*^Including two patients with inferior vena cava tumor thrombi and one patient with concurrent inferior vena cava and right atrial tumor thrombi.

### Treatment efficacy

**Figure 3:**
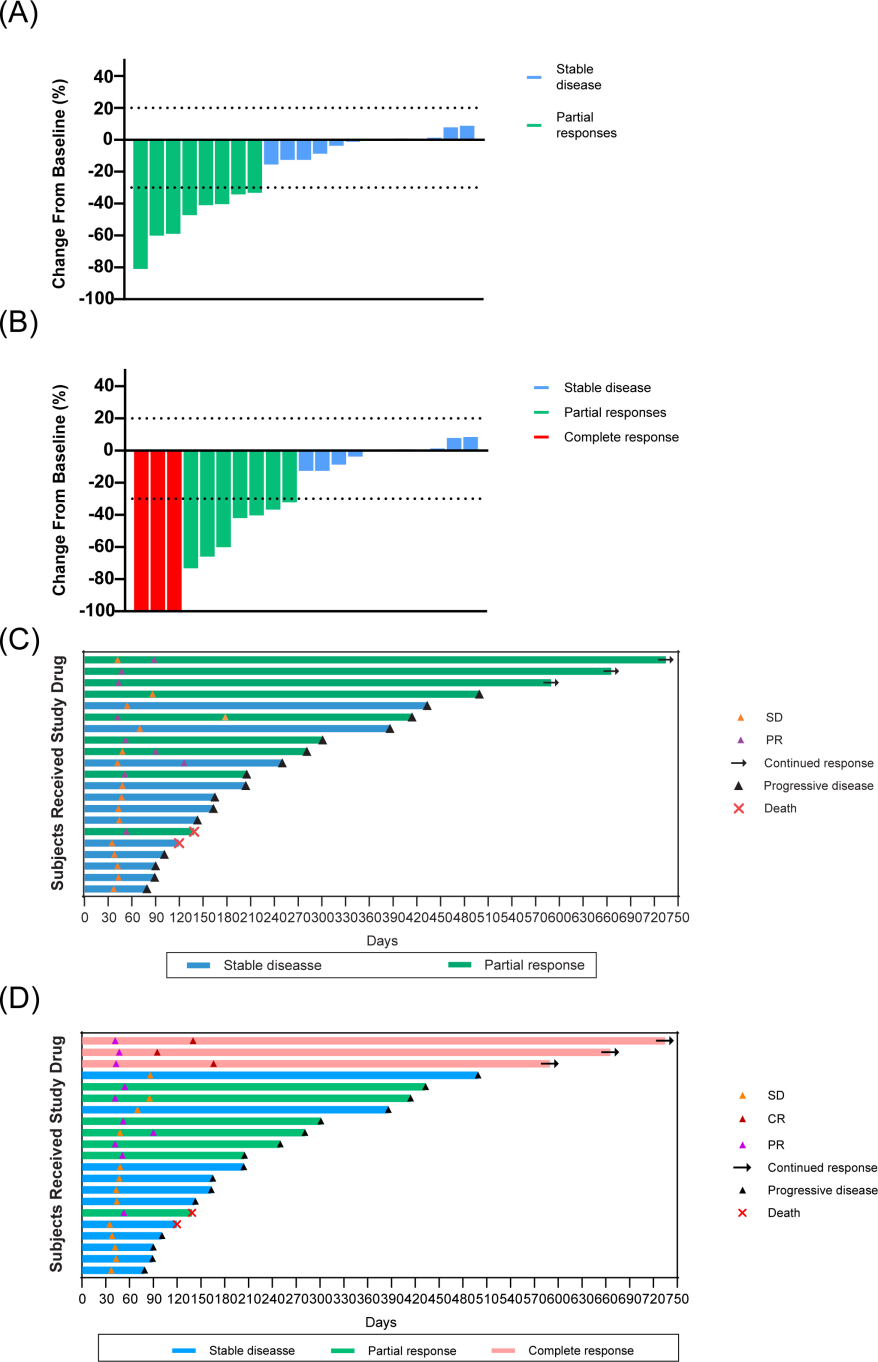
Tumor response characteristics in patients receiving stereotactic body radiation therapy combined with cadonilimab and lenvatinib. **(A)** Maximum percentage reduction from baseline in target lesions of primary tumor(RECIST v1.1 criteria). **(B)** Maximum percentage reduction from baseline in in target lesions of primary tumor (mRECIST criteria). **(C)** Duration of response by RECIST v1.1. **(D)** Duration of response by mRECIST.

**Figure 4:**
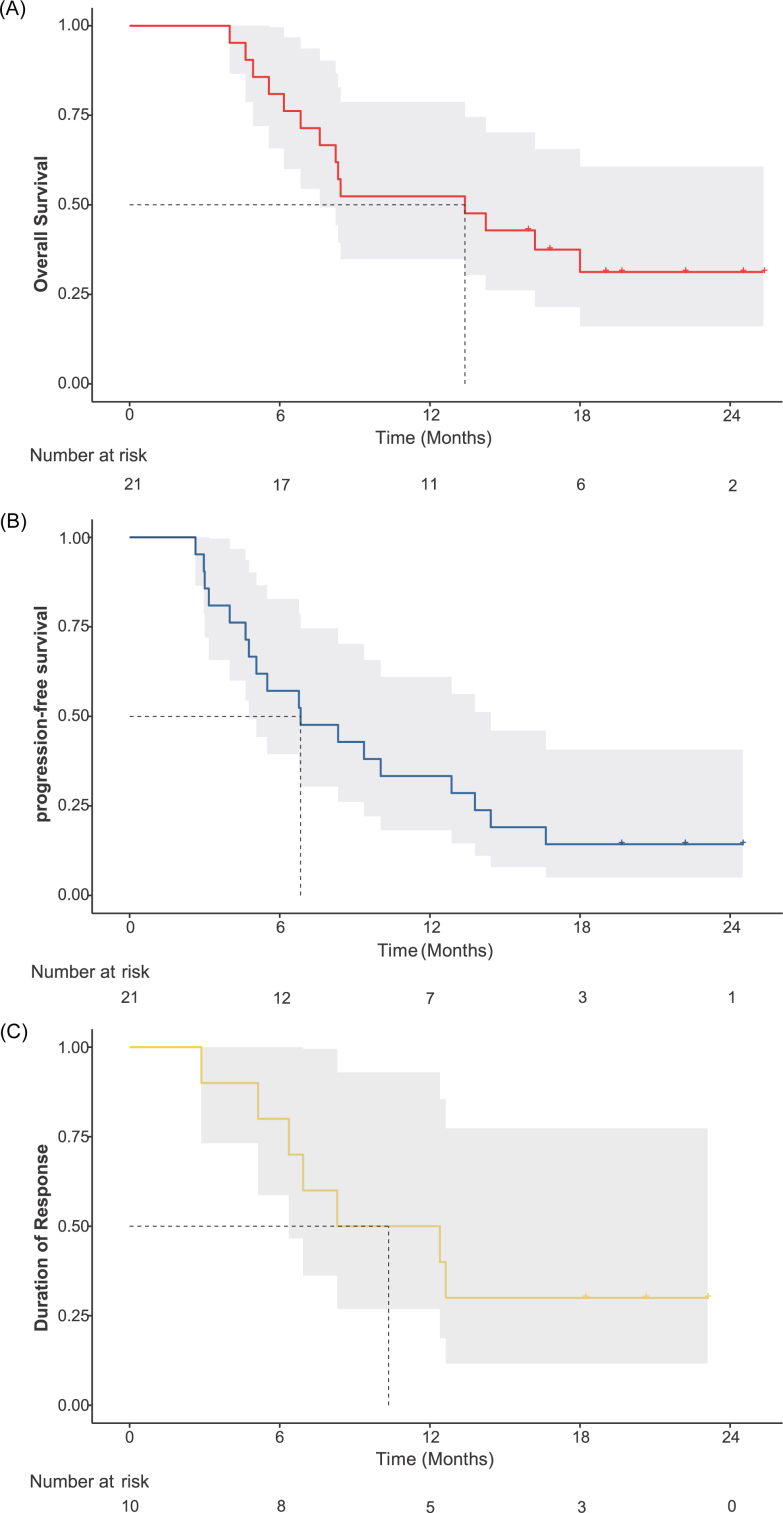
Survival outcomes in the overall study population. Kaplan-Meier curves for **(A)** overall survival (OS), **(B)** progression-free survival (PFS), and **(C)** duration of response (DOR) are shown. NE, not estimable.

**Figure 5:**
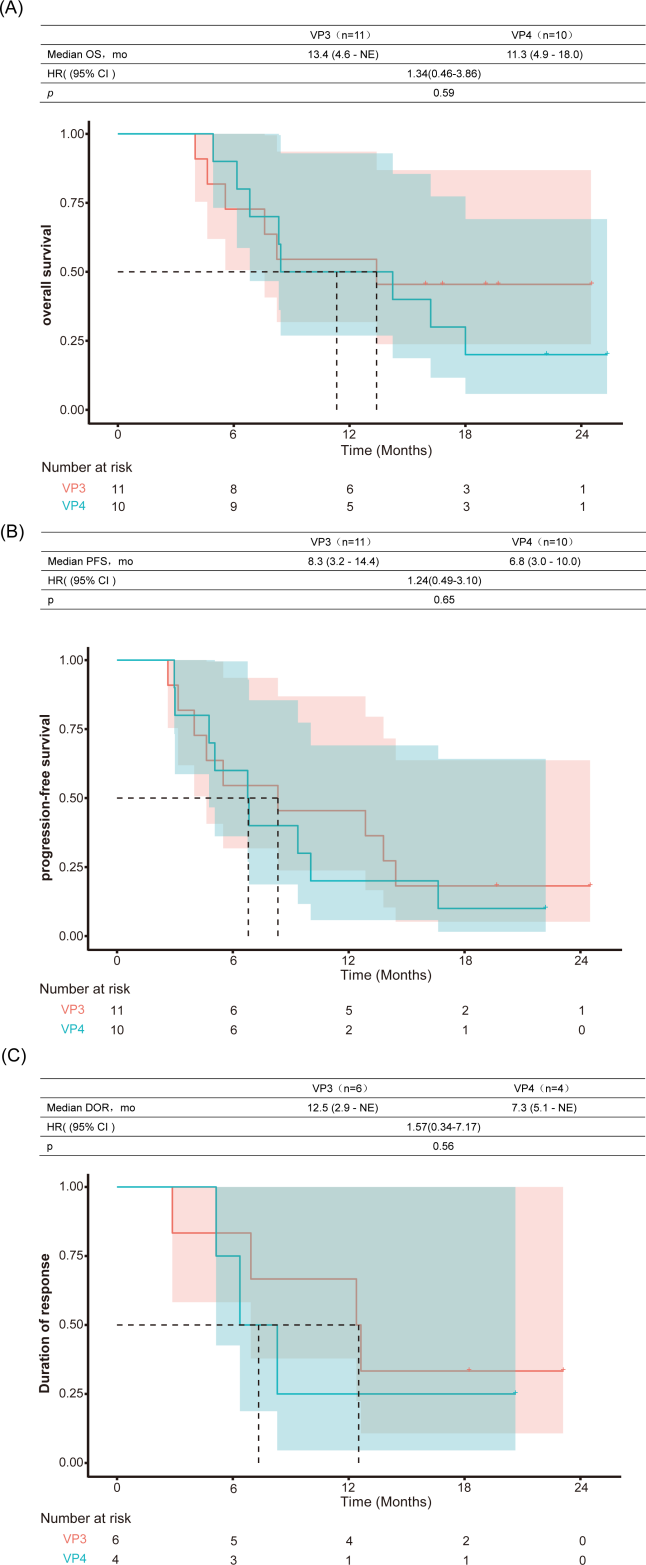
Comparative survival outcomes between Vp3 and Vp4 portal vein tumor thrombosis subgroups. Kaplan-Meier analysis of **(A)** overall survival (OS), **(B)** progression-free survival (PFS), and **(C)** duration of response (DOR). HR, hazard ratio; mo, months; NE, not estimable.

For the evaluation of primary liver tumors across the entire cohort, the ORR was 38.1% and the DCR was 100% according to RECIST version 1.1 criteria (**Table 2**). When assessed using mRECIST criteria, the ORR and DCR were 47.6% and 100%, respectively. Additionally, a subgroup analysis was performed for PVTT subtypes Vp3 and Vp4, with results presented in **Table 2**. In the PVTT assessment, the ORR and DCR were 76.2% and 100%, respectively. On the basis of the RECIST version 1.1 and mRECIST criteria, 14 patients exhibited a reduction in the size of the primary tumor to that at baseline (**Figures 3A**, **B**). Details of the response durations and outcomes are presented in **Figures 3C**, **D**. Among the 21 patients in this study, 14 patients died during follow-up. One surviving patient continued to receive cadonilimab and lenvatinib, two patients discontinued cadonilimab due to financial constraints, and four patients changed their treatment regimen due to tumor progression. The median OS was 13.4 months (95% CI 6.8-not estimable[NE]) for the entire cohort (**Figure 4A**) and 13.4 months (95% CI 4.6-NE) and 11.3 months (95% CI 4.9-18) for Vp3 and Vp4, respectively (p=0.59; **Figure 5A**). The median PFS was 6.8months (95% CI 4.6-12.9)for the entire cohort (**Figure 4B**), 8.3 months (95% CI 3.1-14.4) and 6.8 months (95% CI 3.0-10.0) for Vp3 and Vp4, respectively (p=0.65; **Figure 5B**), and the median time to response (TTR) was 1.7 months. The median DOR was 10.4 months (95% CI 2.9-NE) for the entire cohort (**Figure 4C**), 12.5 months (95% CI 6.9-NE) for the Vp3 group, and 7.3 months (95% CI 5.1-NE) for the Vp4 cohort (p=0.56; **Figure 5C**). The Kaplan–Meier analysis estimated 6-month PFS and OS rates of 57.1% and 81.0%, respectively, and a 12-month OS rate of 52.4% (**Table 2**).

**Table 2:** Best disease responses and survival rate data (n=21).

Variable	Primary liver tumor								PVTT
	RECIST 1.1					mRECIST			
	All (n=21)	Vp3 (n=11)			Vp4 (n=10)	All (n=21)	Vp3 (n=11)	Vp4 (n=10)	
CR-no.(%)	0	0			0	3 (14.3)	2 (9.5)	1 (4.8)	7 (33.3)
PR-no.(%)	8 (38.1)	4 (19.0)			4 (19.0)	7 (33.3)	4 (19.0)	3 (14.3)	9 (42.9)
SD-no.(%)	13 (61.9)	7 (33.3)			6 (28.6)	11 (52.4)	5 (23.8)	6 (28.6)	5 (23.8)
PD-no.(%)	0	0			0	0	0	0	0
ORR-no.(%)	8 (38.1)	4 (19.0)			4 (19.0)	10 (47.6)	6 (28.6)	4 (19.0)	16 (76.2)
DCR-no.(%)	21 (100)	11 (52.4)			10 (47.6)	21 (100)	11 (52.4)	10 (47.6)	21 (100)
Progression-free survival rate (n=21)									
6-month, % (95% CI)		57.1% (95%CI 46.3%-67.9%)						
Overall survival rate (n=21)									
6-month, % (95% CI)		81.0% (95%CI 72.4%-89.6%)					
12-month, % (95% CI)		52.4% (95%CI 41.5%-63.3%)					

CR, complete response; PR, partial response; SD, stable disease; PD, progressive disease; ORR, objective response rate; DCR, disease control rate; PVTT, portal vein tumor thrombus.

### Subgroup analyses of efficacy

#### AFP dynamics and clinical outcomes

**Figure 6:**
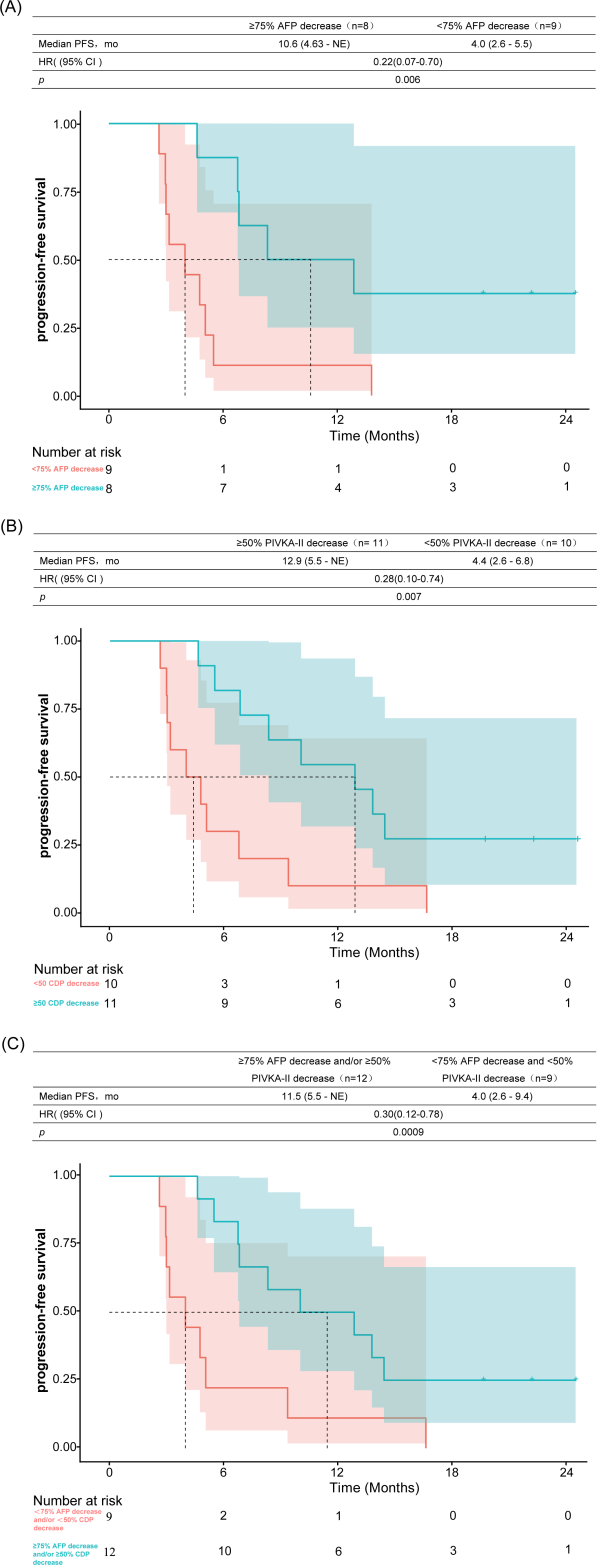
Association between 6-week biomarker response and progression-free survival. Kaplan-Meier curves comparing: **(A)** PFS between patients with ≥ 75% versus <75% AFP reduction; **(B)** PFS between patients with ≥ 50% versus <50% PIVKA-II reduction; **(C)** PFS in dual-responders (≥75% AFP and/or ≥ 50% PIVKA-II reduction) versus non-responders (<75% AFP and <50% PIVKA-II reduction). AFP, α-fetoprotein; PIVKA-II, protein induced by vitamin K absence or antagonist-II; HR, hazard ratio; mo, months; NE, not estimable; PFS, progression-free survival.

**Figure 7:**
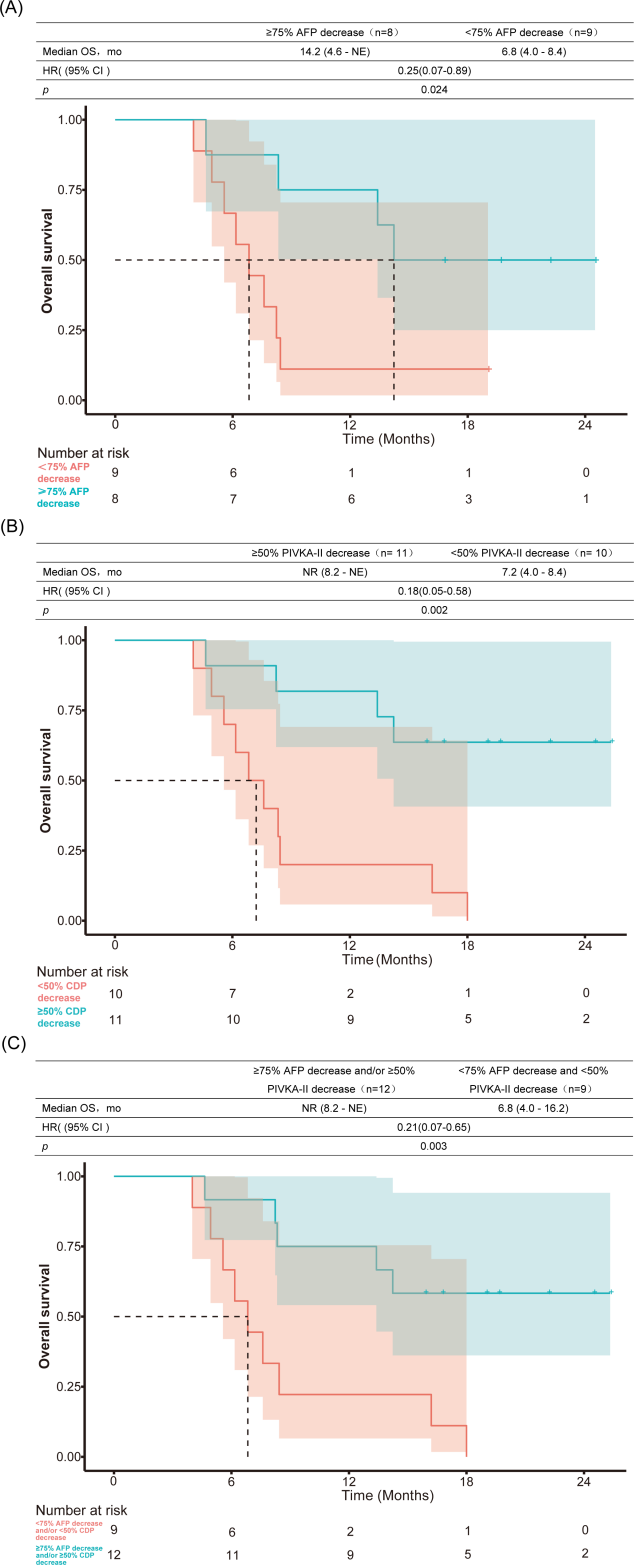
Association between 6-week biomarker response and overall survival. Kaplan-Meier curves comparing: **(A)** OS between patients with ≥ 75% versus <75% AFP reduction; **(B)** OS between patients with ≥ 50% versus <50% PIVKA-II reduction; **(C)** OS in dual-responders (≥75% AFP and/or ≥ 50% PIVKA-II reduction) versus non-responders (<75% AFP and <50% PIVKA-II reduction). AFP, α-fetoprotein; PIVKA-II, protein induced by vitamin K absence or antagonist-II; HR, hazard ratio; mo, months; NE, not estimable; NR, not reached; OS, overall survival.

Among the 21 patients included in the efficacy analysis, 17 (80.9%) had baseline AFP levels >20 ng/mL. Upon re-evaluation two weeks after the initiation of lenvatinib (prior to radiotherapy), the AFP levels decreased in 15 patients (88.2%) and increased in 2 (11.8%). A previous study (32) demonstrated that in patients with baseline AFP levels exceeding 20 ng/mL, a ≥75% reduction at 6 weeks posttreatment initiation was associated with significantly prolonged OS and PFS. Therefore, we reassessed AFP levels at 6 weeks after initiation of systemic therapy, which revealed a decline in 14 patients (66.6%) and increases in 3 patients (14.2%), with a median reduction of 85% (range: -79% to 99%) from baseline. Subgroup analysis revealed that patients who achieved a ≥75% decrease in AFP at 6 weeks had a median PFS of 10.6 months, whereas those with a <75% decrease had a median PFS of 4 months (HR, 0.22; 95% CI: 0.07–0.70; p=0.006; **Figure 6A**). Similarly, the median OS was 14.2 months in the ≥75% AFP-decline group versus 6.8 months in the <75% group (HR, 0.25; 95% CI: 0.07–0.89; p=0.024; **Figure 7A**).

#### PIVKA-II dynamics and clinical outcomes

Baseline PIVKA-II levels were markedly elevated (>2050 mAU/mL) in 19 patients (90.5%), while relatively lower levels (113 and 1056 mAU/mL, respectively) were observed in the remaining two patients Following a 2-week course of lenvatinib therapy, we observed divergent PIVKA-II trajectories: 8 patients (38.1%) exhibited declining levels, whereas 13 patients (61.9%) presented increasing values prior to radiotherapy. The classification of PIVKA-II response was based on a prior study (33) involving 235 patients with HCC receiving PD-1 blockade therapy, which demonstrated that a >50% reduction in PIVKA-II levels was significantly associated with prolonged PFS (p=0.021) and OS (p=0.006). We re-evaluated PIVKA-II levels at 6 weeks after the initiation of systemic therapy. Subgroup analysis revealed significant differences in median PFS and OS between the PIVKA-II response groups. The median PFS was 12.9 months in the ≥50% PIVKA-II decline group compared with 4.4 months in the <50% PIVKA-II decline group (HR, 0.28; 95% CI: 0.10–0.74; p=0.007; **Figure 6B**). Similarly, the median OS was not reached in the ≥50% PIVKA-II decline group versus 7.2 months in the <50% decline group (HR, 0.18; 95% CI: 0.05–0.58; p=0.002; **Figure 7B**).

Further analysis categorized patients into two groups; those with a ≥ 75% AFP decrease and/or ≥ 50% PIVKA-II decrease and those with a <75% AFP decrease and <50% PIVKA-II decrease (the latter included two patients with normal baseline AFP levels who also exhibited a <50% PIVKA-II decrease after treatment). Compared with the control group, the group with ≥ 75% AFP decrease and/or ≥ 50% PIVKA-II decrease had significantly longer PFS (11.5 vs. 4.0 months, HR, 0.30; 95% CI: 0.12–0.78; p=0.0009; **Figure 6C**) and OS (non-reached vs. 6.8 months, HR, 0.21; 95% CI: 0.07–0.65; p=0.003; **Figure 7C**).

### Safety

TRAEs were evaluated on the basis of their frequency and severity using CTCAE version 5.0. As shown in **Table 3**, the most common TRAEs of any grade were hypertension (50%), thrombocytopenia (42%), and fatigue (38%). The most frequent grade 3/4 TRAEs were hypertension (17%), fatigue (8%), AST elevation (8%), and immune-mediated dermatitis (8%). None of the patients developed classical radiation-induced liver disease (RILD). Two patients discontinued treatment after one cycle of cadonilimab due to treatment-emergent adverse events (TEAEs) (**Figure 2**): one experienced a cerebral infarction, and the other developed upper gastrointestinal bleeding. Additionally, four patients temporarily stopped treatment during the medication period because of immune-related adverse events (irAEs), including two cases of immune-mediated dermatitis, one case of immune-mediated hepatitis, and one case of immune-mediated enteritis. Following active symptomatic management, all irAEs resolved sufficiently to allow patients to resume their original treatment regimen. During the follow-up period, one patient succumbed to COVID-19-related complications at a local hospital, whereas an unexplained out-of-hospital death occurred in another patient before progression of disease (**Figures 2**, **3C**, **D**); no deaths were considered related to the study treatment.

**Table 3:** Treatment related adverse events (n=24).

Adverse events	Any grade, n (%)	Grade 3/4. n (%)
Any adverse event	22 (92)	10 (42)
Fatigue	9 (38)	2 (8)
Rash	5 (21)	1 (4)
Anorexia	4 (17)	0
Hypertension	12 (50)	4 (17)
Proteinuria	4 (17)	0
leukopenia	6 (25)	1 (4)
Neutropenia	3 (13)	1 (4)
Thrombocytopenia	10 (42)	0
AST elevation	8 (33)	2 (8)
ALT elevation	4 (17)	1 (4)
Hyperbilirubinemia	5 (21)	0
Infusion related reaction	1 (4)	0
Palmar-plantar arerythrodysesthesia syndrome	1 (4)	0
Gastric ulcer	2 (8)	0
Adrenal insufficiency	4 (17)	0
Immune-mediated pneumonitis	1 (4)	0
Immune-mediated colitis	0	1 (4)
Immune-mediated dermatitis	5 (21)	2 (8)
Immune-mediated hepatitis	1 (4)	1 (4)

## Discussion

Portal vein involvement in HCC is a marker of advanced disease and usually indicates a poor prognosis, particularly in the Vp3/4 patient population. Recent studies have demonstrated the promising efficacy of combined locoregional and systemic therapies in improving clinical outcomes for patients with advanced HCC and PVTT (34, 35). However, the optimal combination therapy for patients with HCC and PVTT is not well defined. In this study, we assessed the clinical benefits of triple therapy combining cadonilimab, lenvatinib, and SBRT for HCC patients with Vp3/4. The tumor ORR was 38.1% according to RECIST version 1.1 and 47.6% according to the mRECIST criteria. The median PFS and OS were 6.8months and 13.4 months, respectively. To our knowledge, this study represents the first prospective clinical trial to evaluate the efficacy and safety of sequential treatment with lenvatinib, followed by SBRT and the bispecific antibody (PD-1/CTLA-4) cadonilimab in patients with PVTT. Our findings suggest the promising efficacy and durable response of this combination regimen. Furthermore, the toxicity was manageable, with no unexpected safety signals identified.

Patients with HCC complicated by PVTT frequently face increased risks of ascites, jaundice, abnormal liver function, intrahepatic metastasis, and distant metastasis due to portal vein reflux obstruction, which significantly impacts treatment outcomes. Therefore, early and rapid relief of the tumor thrombus represents a critical breakthrough in treating these patients, providing them with additional therapeutic options. Recent studies have demonstrated that PVTT is more sensitive to radiotherapy than are primary HCC lesions (30, 36–38). However, patients with Vp3/4 PVTT often exhibit poor liver function reserves. Combining radiotherapy with targeted immunotherapy for intrahepatic tumors may increase the risk of liver function impairment, potentially leading to intolerance of subsequent treatments and affecting OS. Consequently, this study focused on radiotherapy targeting the PVTT aiming to achieve rapid cytoreduction of the neoplastic burden, reduce the degree of hemodynamic compromise associated with portal hypertension, and restore physiological portal venous flow while preserving hepatic functional reserve through precision dosimetry. This therapeutic rationale was designed to balance oncological efficacy with organ preservation in patients with advanced HCC. Additionally, controlling PVTT effectively eliminates tumor cell invasion into the local blood flow, which reduces further metastasis, ultimately yielding encouraging tumor responses and survival outcomes (36, 39).The findings of this study are consistent with previously published results (20) and support the efficacy of SBRT combined with targeted and immunotherapy for hepatocellular carcinoma with portal vein tumor thrombus.

Given the high tumor burden and frequent occurrence of distant metastases in this cohort, rapid systemic disease control was imperative. In the treatment plan design, lenvatinib was administered orally for 2 weeks prior to radiotherapy, followed by the first cycle of cadonilimab within 4 ± 3 days postradiotherapy. The theoretical foundation of this study design stemmed from research indicating that antiangiogenic drugs enhance radiotherapy efficacy by normalizing tumor blood vessels and creating an immune-friendly tumor microenvironment (TME) (40). Radiotherapy further promotes the infiltration of immune cells, particularly cytotoxic CD8+ T cells, into the TME (24). Furthermore, radiotherapy may induce the expression of immune checkpoint molecules (e.g., PD-1, PD-L1, and CTLA-4) and vascular endothelial growth factor (VEGF) (24). The combination of immune checkpoint blockade (ICB) with radiotherapy and anti-VEGF therapy restores and enhances antitumor immunity, thereby improving therapeutic efficacy. We observed that 88.2% of patients exhibited decreased AFP levels after 2 weeks of lenvatinib treatment, suggesting an early therapeutic effect of lenvatinib. In the context of targeted therapies (including sorafenib and lenvatinib) and immunotherapeutic agents, patients with significant AFP reduction typically have a higher ORR and prolonged survival (41, 42). Consistently, our subgroup analysis demonstrated that achieving a ≥ 75% reduction in AFP levels at 6 weeks posttreatment initiation was significantly associated with prolonged OS and PFS. These findings align with previous research investigating AFP as a potential surrogate biomarker in HCC treatment (32), further corroborate the utility of the AFP level as an early indicator of therapeutic response. Our study also revealed that treatment-induced PIVKA-II reduction ≥ 50% was associated with significantly better clinical outcomes, conferring both prolonged PFS and OS compared with a suboptimal biomarker response. However, the current evidence remains inconclusive regarding optimal threshold values for posttreatment reductions in either AFP or PIVKA-II in predicting HCC treatment efficacy. Varying cutoffs have been proposed, including a ≥ 40% AFP reduction at 1 month postlenvatinib initiation in a study that demonstrated 100% sensitivity and 78% specificity in distinguishing patients who achieved disease control from primary progressors (43). Similarly, Chen et al. reported that a ≥ 50% reduction in PIVKA-II levels was correlated with improved PFS and OS in patients across various HCC treatment modalities (44). While the study provided valuable insights, notably, the enrolled population exclusively comprised patients with PIVKA-II-secreting tumors. In clinical practice, most patients with HCC exhibit both AFP- and PIVKA-II-secreting tumors, as reflected in our cohort. Our results are supported by Sun et al.’s study of 235 patients with HCC treated with programmed cell death-1 blockade therapy, where ≥ 50% reductions in both PIVKA-II and AFP levels significantly correlated with longer PFS (p<0.001 and p=0.021, respectively) and OS (p=0.003 and p=0.006, respectively) (33). Our findings further substantiate the clinical value of combined AFP and PIVKA-II analysis, demonstrating that a ≥ 75% decrease in AFP and/or a ≥ 50% decrease in PIVKA-II was associated with improved PFS (p=0.009) and OS (p=0.003). Nevertheless, given the current paucity of robust evidence regarding the predictive value of these biomarkers in management of HCC, validation through large-scale, multicenter cohort studies is imperative to establish their definitive prognostic utility.

The patients with HCC in this study presented a relatively large tumor burden, and 50% were complicated by Vp4 PVTT, including two patients with inferior vena cava tumor thrombi and one patient with both inferior vena cava and right atrium tumor thrombi. Sixty-two percent of patients had a maximum tumor diameter ≥ 10 cm, 81.0% had multiple tumor lesions, and 48% had extrahepatic metastasis. The median PFS and OS for the patients were 6.8 months and 13.4 months, respectively, surpassing the 5.4 months and 7.6 months reported in the IMbrave 150 study (7). However, considering the small sample size and inclusion of some Vp3 patients, it is premature to conclude that this treatment regimen outperforms IMbrave 150. Nevertheless, compared with a recently published multicenter, open-label, noncontrolled, randomized trial (20), the OS and PFS achieved in this study were superior to those of camrelizumab plus apatinib and SBRT (mOS 13.4 vs. 12.7 months; mPFS 8.3 vs. 4.6 months). The improved long-term efficacy observed in our study might be attributed to the use of cadonilimab. PD-1 monoclonal antibodies act primarily on peripheral T cells to relieve immunosuppression, whereas CTLA-4 monoclonal antibodies predominantly target the initial activation phase of T cells. This distinct mechanism of action not only limits synergistic effects but also may lead to overlapping side effects, thereby increasing the incidence of irAEs. The innovation of cadonilimab lies in combining PD-1 and CTLA-4 monoclonal antibodies into a single highly effective bispecific antibody (45). Its higher avidity in a novel tetravalent format may enhance drug retention. Moreover, the Fc-null design eliminates Fc receptor-mediated effector functions, reducing the release of proinflammatory cytokines that can deplete PD-1-expressing T cells and compromise antitumor efficacy. In addition, this design reduces the risk of side effects, leading to more significant treatment benefits for patients. Another recent prospective trial (46) assessed the efficacy and safety of intensity-modulated radiotherapy (IMRT) combined with systemic atezolizumab and bevacizumab in 30 patients with HCC accompanied by extrahepatic PVTT. The ORR for the entire cohort was 76.6%, with a median OS of 9.8 months and a median PFS of 8.0 months, respectively. Although cross-trial comparisons should be interpreted with caution, the observed ORR advantage of the study did not correlate with significant OS improvements. Of particular concern, the study documented two cases of grade ≥ 3 gastrointestinal bleeding events, representing a higher incidence than previously reported for atezolizumab/bevacizumab monotherapy (7, 47). The authors speculated that this might be due to most patients included in the study having Vp4 PVTT (48). Alternatively, patients with Vp4 PVTT may benefit more from lenvatinib, which is associated with a lower risk of gastrointestinal bleeding. Notably, the combination of cadonilimab, lenvatinib, and SBRT achieved the longest OS in recently published prospective studies involving Vp3/4 HCC. Our findings are consistent with those of a previously published clinical study (15), which demonstrated that the combination of lenvatinib and cadonilimab yielded higher response rates and prolonged OS in patients with advanced HCC. This may be attributed to the use of lenvatinib as an antiangiogenic agent, which has a lower incidence of gastrointestinal bleeding than bevacizumab does. Additionally, dual antibody treatment with cadonilimab may prolong the development of drug resistance. Immunotherapy has been shown to provide durable and sustained responses, as evidenced by the significant survival tail in the KM curve of OS in this study. These findings suggest that HCC cells are sensitive to CTLA-4/PD-1/PD-L1 blockade, which is consistent with prior observations with combinations such as nivolumab plus ipilimumab (12) and tremelimumab plus durvalumab (11).

In this study, the safety profile of the regimen used revealed no new or unexpected toxicity. Among the common adverse reactions, adrenal insufficiency and immune-mediated dermatitis were predominantly associated with cadonilimab, whereas hypertension, leukopenia, and proteinuria were more frequently linked to lenvatinib. The incidence of grade ≥ 3TRAEs was 42%, comparable to the 41% rate observed with first-line ipilimumab-nivolumab combination therapy in the CheckMate 9DW trial (12). The most frequent grade ≥ 3 TRAEs was hypertension (17%), which is consistent with the known toxicity profile of lenvatinib. Consequently, the incidence of ≥ 3 irAEs in our study cohort was lower than that observed with the O+Y regimen, further corroborating the favorable irAE profile associated with cadonilimab monotherapy. However, the incidence of ≥ 3 irAEs in our study was higher than that reported previously for the cadonilimab/lenvatinib combination in patients with advanced HCC (15). The observed discrepancies between our findings and those of prior trials may be attributed to distinct baseline patient characteristics. Notably, our cohort included a greater proportion of patients with advanced disease manifestations, including Vp4 PVTT (48%) and concurrent inferior vena cava/right atrial tumor embolus (14%) populations systematically excluded in prior trials (15). These subgroups are associated with increased tumor aggressiveness, more immunosuppressive TMEs, and compromised hepatic reserves, all of which may collectively contribute to reduced treatment responsiveness and increased susceptibility to AEs. These adverse effects are generally manageable through medication or dose adjustments and are typically not life-threatening. There were no treatment-related deaths. Additionally, no cases of RILD were observed, likely due to the precision of SBRT in sparing healthy tissues (49).

This study had several limitations. First, although it was conducted across multiple centers, the number of patients involved was relatively small, which may have influenced the robustness of the findings. Second, this was a single-arm study without a control group, making it challenging to fully evaluate the benefits of this triple therapy for patients with unresectable HCC and PVTT. Third, given the high prevalence of HBV infection in China and the absence of international regulatory approval for cadonilimab, the generalizability of our findings to other populations may be limited. Thus, our results should be interpreted cautiously and require validation through larger, randomized controlled trials.

## Conclusion

In summary, our findings demonstrate that the combination of SBRT with cadonilimab and lenvatinib exhibits promising therapeutic efficacy and a manageable safety profile, suggesting its potential as a viable treatment option for HCC patients with Vp3 or Vp4 PVTT. Early AFP or PIVKA-II response at 6 weeks may serve as a prognostic biomarker.

## Data Availability

The original contributions presented in the study are included in the article/supplementary material. Further inquiries can be directed to the corresponding author/s.
